# Cerebral Small Vessel Disease, Hypertension, and Vascular Contributions to Cognitive Impairment and Dementia

**DOI:** 10.1161/HYPERTENSIONAHA.123.19943

**Published:** 2023-11-29

**Authors:** Atticus H. Hainsworth, Hugh S. Markus, Julie A. Schneider

**Affiliations:** Molecular and Clinical Sciences Research Institute, St George’s University of London, United Kingdom (A.H.H.).; Department of Neurology, St George’s University Hospitals NHS Foundation Trust, London, United Kingdom (A.H.H.).; Stroke Research Group, Department of Clinical Neurosciences, University of Cambridge, United Kingdom (H.S.M.).; Rush Alzheimer’s Disease Center, Departments of Pathology and Neurological Sciences, Rush University Medical Center, Chicago, IL (J.A.S.).

**Keywords:** arteries, blood pressure, dementia, genetics, neuropathology, stroke, white matter

## Abstract

Hypertension-associated cerebral small vessel disease is a common finding in older people. Strongly associated with age and hypertension, small vessel disease is found at autopsy in over 50% of people aged ≥65 years, with a spectrum of clinical manifestations. It is the main cause of lacunar stroke and a major source of vascular contributions to cognitive impairment and dementia. The brain areas affected are subcortical and periventricular white matter and deep gray nuclei. Neuropathological sequelae are diffuse white matter lesions (seen as white matter hyperintensities on T2-weighted magnetic resonance imaging), small ischemic foci (lacunes or microinfarcts), and less commonly, subcortical microhemorrhages. The most common form of cerebral small vessel disease is concentric, fibrotic thickening of small penetrating arteries (up to 300 microns outer diameter) termed arteriolosclerosis. Less common forms are small artery atheroma and lipohyalinosis (the lesions described by C. Miller Fisher adjacent to lacunes). Other microvascular lesions that are not reviewed here include cerebral amyloid angiopathy and venous collagenosis. Here, we review the epidemiology, neuropathology, clinical management, genetics, preclinical models, and pathogenesis of hypertensive small vessel disease. Knowledge gaps include initiating factors, molecular pathogenesis, relationships between arterial pathology and tissue damage, possible reversibility, pharmacological targets, and molecular biomarkers. Progress is anticipated from multicell transcriptomic and proteomic profiling, novel experimental models and further target-finding and interventional clinical studies.

Cerebral small vessel disease (SVD) is the term used to describe a brain microvascular disease or syndrome that is common in older people and, depending on the definition, occurs in ≈50% of people aged over 65 years.^1–4^ SVD is considered to be the primary cause of lacunar strokes.^5–8^ It is also a major factor in vascular contributions to cognitive impairment and dementia (VCID).^8–11^

In clinical practice, SVD is diagnosed on the basis of hallmark radiological features seen on magnetic resonance imaging (MRI, eg, Figure 1)^8,12^: (1) subcortical small, focal infarcts, (2) diffuse white matter lesions, seen as white matter hyperintensities (WMH) on T2-weighted images, (3) less commonly, microhemorrhages in subcortical areas. Most (>95%) SVD is nonheritable, or sporadic, although over 50 genetic loci associated with the risk of sporadic SVD have emerged from genome-wide association studies in recent years.^13–15^ Heritable, monogenic forms of SVD include cerebral autosomal dominant arteriopathy with subcortical infarcts and leukoencephalopathy (CADASIL; due to mutations in *NOTCH3*), CARASIL (*HTRA1*), and collagen-IV (*COL4A1/COL4A2*).^14^ Relative to the prevalent, sporadic SVD, these are rare, less strongly associated with hypertension and detected clinically in younger people, usually with more severe disease.

**Figure 1.**
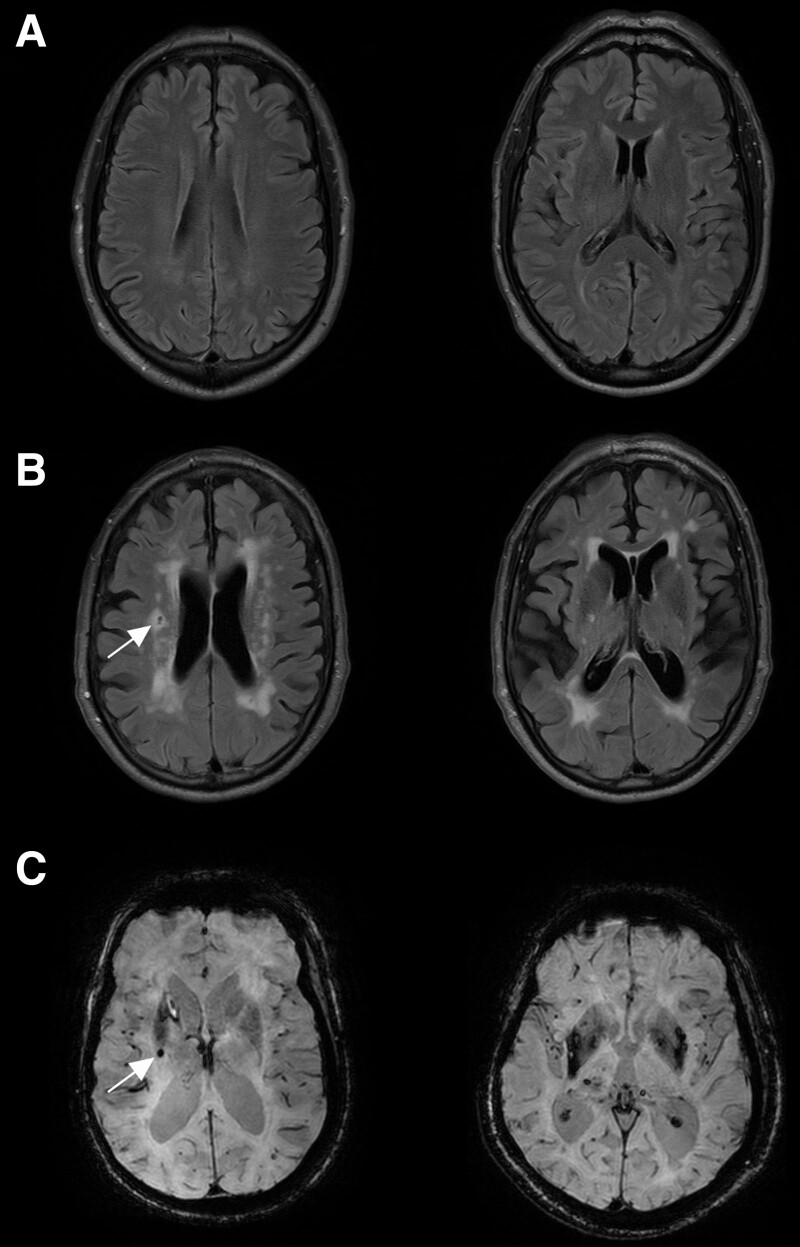
**Magnetic resonance imaging (MRI) evidence of small vessel disease (SVD). A**, Fluid-attenuated inversion recovery (FLAIR) MRI scans of an older patient with minimal white matter hyperintensities. **B**, FLAIR MRI scans from equivalent areas of another older patient with extensive white matter hyperintensities and a cavitated lacune (arrow). **C**, Susceptibility-weighted images from 2 different patients with SVD, showing multiple microbleeds (eg, marked with an arrow).

This review covers the sporadic forms of SVD that are associated with hypertension. In practice, this means 3 well-defined neuropathological lesions in cerebral small arteries: arteriolosclerosis, lipohyalinosis, and microatheroma (eg, Figure 2). Other forms of brain microvascular disease have been reviewed elsewhere.^8,16,17^ Here, we will summarize the epidemiology, neuropathology, clinical management, genetics, and preclinical models of sporadic hypertensive SVD, ending with an overview of theories about pathogenesis.

**Figure 2.**
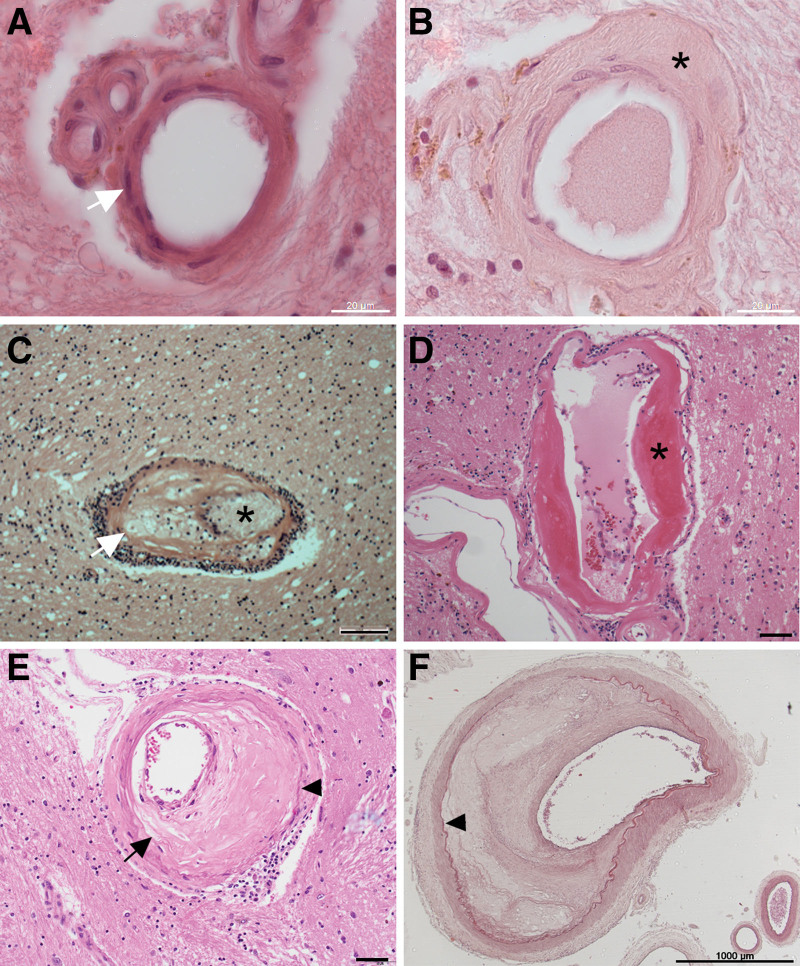
**Small arterial vessels in human brain: examples of hypertensive small vessel disease. A**, A normal, healthy penetrating artery with thin wall and multiple myocyte nuclei (eg, marked with arrow). **B**, Arteriolosclerosis, with approximately concentric wall thickening, with acellular hyaline material (asterisk). **C**, Lipohyalinosis, with asymmetrical wall thickening around the lumen (*) and mural lipid-containing macrophages (arrow). **D**, Fibrinoid necrosis in a larger penetrating vessel. The wall is asymmetrically thickened with eosinophilic fibrinoid material (*). **E**, Microatheroma in a penetrating vessel. Asymmetrical thickening and cholesterol clefts are seen as open slits (arrow) in the intimal layer. Internal lamina is visible (arrowhead) delimiting the intima. **F**, For comparison, large vessel atheroma in a leptomeningeal artery, with internal lamina clearly visible (arrowhead). Hematoxylin and eosin staining. Scale bars: **A** and **B**, 20 microns, **C** through **E**, 50 microns, **F**, 1000 microns. **C** and **D** were kindly supplied by Professor Colin Smith, University of Edinburgh.

## NOMENCLATURE

The nomenclature of SVD is confusing, often inexact and varies across subspecialties. SVD is used to refer to a myriad of small vessel abnormalities with or without downstream tissue injuries.^4,8,18^ In diagnostic neuropathology, SVD often refers to arteriolosclerosis and associated tissue lesions. In clinical practice, usage is usually limited to the tissue injury aspect, which can be visualized radiologically and is presumed due to brain microvascular disease (which can only be visualized under a microscope). More recently, enlarged perivascular spaces and microbleeds are sometimes included in the rubric. For the purposes of this review, the term SVD refers to hypertension-associated sporadic forms of brain microvascular disease unless stated otherwise.

## METHODS

This review is based on the authors’ knowledge, with expertise related to SVD in cellular and in vivo neuroscience (A.H.H.), clinical management, imaging and genetics (H.S.M.), neuropathology, and epidemiology (J.A.S.). References are derived from the authors’ archives and PubMed searches.

## EPIDEMIOLOGY OF SVD AND VCID

### ROSMAP Cohorts

Because SVD nomenclature varies across disciplines and the underlying vessel pathologies are only visualized on brain tissue, the prevalence of SVD is difficult to estimate with certainty. The Religious Orders Study and Rush Memory and Aging Project (ROSMAP) are large clinicopathologic studies of older people enrolled without known dementia, with annual clinical assessments and agreement to brain donation at death (Table 1). In the ROSMAP studies, with over 1700 donated brains, the severity of arteriolosclerosis is determined at the level of the anterior basal ganglia. With an average age of death at about 90 years, SVD is highly prevalent, with about one-third of the cohort showing moderate-to-severe arteriolosclerosis.^3,11,29^ Prevalence of hypertension in ROSMAP is 67% to 69% with some variation as the cohort grows.^3^ ROSMAP participants are predominantly White (90%–95%).

**Table 1.**
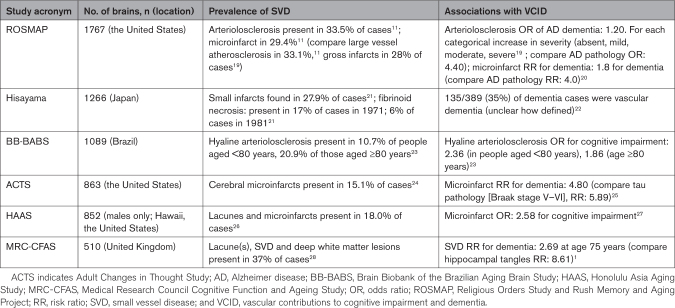
Prevalence of SVD and Associations With VCID in Large Autopsy Series (N=500 or More Brains)

### SVD Epidemiology

In the ROSMAP cohort, arteriolosclerosis is most significantly associated with elevated blood pressure and age.^30^ Arteriolosclerosis is more common in women, but this is difficult to dissociate from longevity in women.^31^ Severity of arteriolosclerosis has been noted to be more severe in African Americans.^32^ Microvascular disease is common in people with Alzheimer disease (AD) pathology, and the association with AD and other neurodegenerative pathologies may be complex. Arteriolosclerosis is related to Alzheimer type dementia but this may be partly through the associated and added tissue injury in the parenchyma.^33^

Many people with arteriolosclerosis also have large vessel atherosclerosis, which is also common in older people. About 30% of older people have moderate-to-severe large vessel atherosclerosis.^11^ Similar to arteriolosclerosis, both age and blood pressure are strong risk factors for atherosclerosis, and downstream consequences can include various tissue injuries, including white matter changes and infarct (the latter often larger than in arteriolosclerosis). While definitions of SVD pathology vary, similar patterns of prevalence have been seen in other large cohorts^1,27^ (Table 1).

### VCID Epidemiology

Blood pressure is an established risk factor for dementia, with risk accruing in midlife not just in old age. Hypertension contributes to cognitive impairment through pathways independent of symptomatic stroke. This extensive topic is well covered in recent reviews.^34,35^

Arteriolosclerosis is consistently related to dementia in older people. Although strongly associated with hypertension, arteriolosclerosis is not exclusively seen in hypertension and is also seen in aging and diabetes.^36^ One of the pathways by which arteriolosclerosis may contribute to dementia is via small microinfarcts in the aging brain.^37^ In a pathway analysis of the neuropathological features that mediate the association of age with dementia in the ROSMAP cohort, about 30% of the association was related to vascular pathways.^38^

Most studies have shown that the contribution of SVD to cognitive impairment is additive, rather than synergistic, with that of AD pathology (amyloid and tangles).^26,39^ Even after accounting for infarcts, arteriolosclerosis has an added contribution to dementia and to multiple domains of cognitive impairment.^40^

## NEUROPATHOLOGY OF SVD

### Vessel Pathology

The 3 main forms of hypertensive SVD are compared in Table 2 (see examples in Figure 2). In the landmark serial sectioning studies of Fisher^5,45–47^, 2 vascular lesions were identified upstream from lacunes: lipohyalinosis and atheroma in small penetrating arteries (microatheroma). These 2 are now rarely seen in diagnostic neuropathology or in brain autopsy series. This may be due to the widespread use of blood pressure medications,^36^ or the fact that we do not deliberately sample them. Still common is a third lesion, seen in the smallest penetrating arteries (up to 0.3 mm outer diameter), termed arteriolar sclerosis or arteriolosclerosis (small artery stiffening).^6,7,36^ It appears likely that Fisher and some earlier neuropathologists, who did not have access to MRI, immunohistochemistry, or genetic data, considered arteriolosclerosis a part of normal brain aging.^41,47,49^ More recent brain series have associated arteriolosclerosis with hypertension,^36,43^ WMH load,^44,50^ incidence of lacunar stroke,^43^ VCID and dementia.^9–11^

**Table 2.**
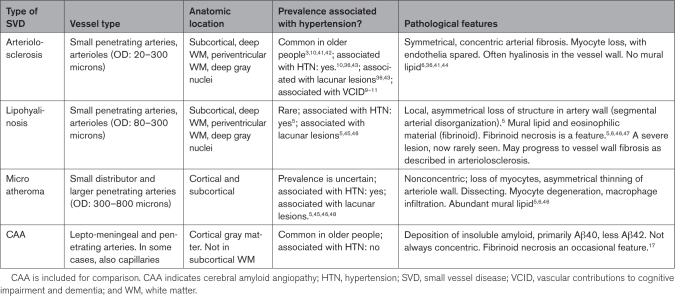
Comparing Different Neuropathological Forms of Hypertensive SVD

#### Lipohyalinosis

Lipohyalinosis was identified by Fisher^5,46^ as the causal lesion upstream from 40 of 50 lacunes, in brains from 4 people with severe hypertension. Lipohyalinosis describes small arteries whose walls exhibited loss of myocytes, fibrosis, and fatty macrophages with lipid deposits^5^ (Figure 2C). The lesion is eccentric, extending along a short segment of the vessel wall (hence the term segmental disorganization).^5,47^ The wall contains an eosinophilic deposit termed fibrinoid.^5,51^ Fibrinoid necrosis in the vessel wall (Figure 2D) is a feature of lipohyalinosis, found most commonly in uncontrolled or malignant hypertension. Fibrinoid necrosis may be a transitory status for the vessel. The weakening of the wall may result in a Charcot-Bouchard aneurysm, with or without hemorrhage. Although originally thought to be specific to hypertension,^5^ lipohyalinosis is also (uncommonly) seen in the brains of people without hypertension.^36^ Lipohyalinosis is considered a more acute or severe form of SVD that has been suggested to transition into end-stage fibrosis or arteriolosclerosis.

#### Microatheroma

Microatheroma refers to atherosclerosis in larger small arteries (approximate range, 0.3–0.8 mm outer diameter) analogous to that frequently detected in large arteries (such as the carotids or middle cerebral artery) of older people with hypertension. It features local, eccentric lipid deposits in the vessel wall, with myocyte loss and fatty macrophages (foam cells) in the wall^5,6^ (Figure 2E). Fibrinoid necrosis is not a feature. Microatheroma in the parent vessel at the origin of the perforating artery, or in the proximal larger perforator arteries themselves, has been associated with lacunar stroke.^5,6,46^ It is usually associated with larger lacunar stroke, often isolated, and without prominent WMH.^48^ Microatheroma has been associated with an atherogenic risk factor profile, in contrast with the predominant role of hypertension in arteriolosclerosis.

Although microatheroma is now a rare finding in diagnostic neuropathology (at least in higher–income countries), we cannot assume it is absent. Intracranial atherosclerotic stenosis was detected in 45% of a large, older cohort in a recent magnetic resonance angiography imaging study.^52^ Microatheroma favors arterial branch points, for example, at the perpendicular origin of MCA-derived perforators, a location rarely sampled in a histopathology block. It seems reasonable to assume, as Fisher did, that an individual with severe large vessel atheroma (Figure 2F) has a high likelihood of microatheroma. A dedicated histological study to assess microatheroma as a risk factor for human stroke, SVD, and VCID would be valuable.

A belief persists that microatheroma is more common in some racial groups, specifically African Americans (see a classic study by Caplan et al^53^, and early references therein). While there is heterogeneous evidence around this concept, non-white brain archives will allow it to be tested.^54^

#### Arteriolosclerosis

Arteriolosclerosis is a fibrous, hyaline thickening of small penetrating arteries in subcortical areas (subcortical and periventricular white matter and deep gray nuclei). Hypertension and old age are strong risk factors, with poor glucose control, elevated plasma homocysteine, and tobacco smoking also implicated. Links to hyperlipidemia are less well evidenced.

**Figure 3.**
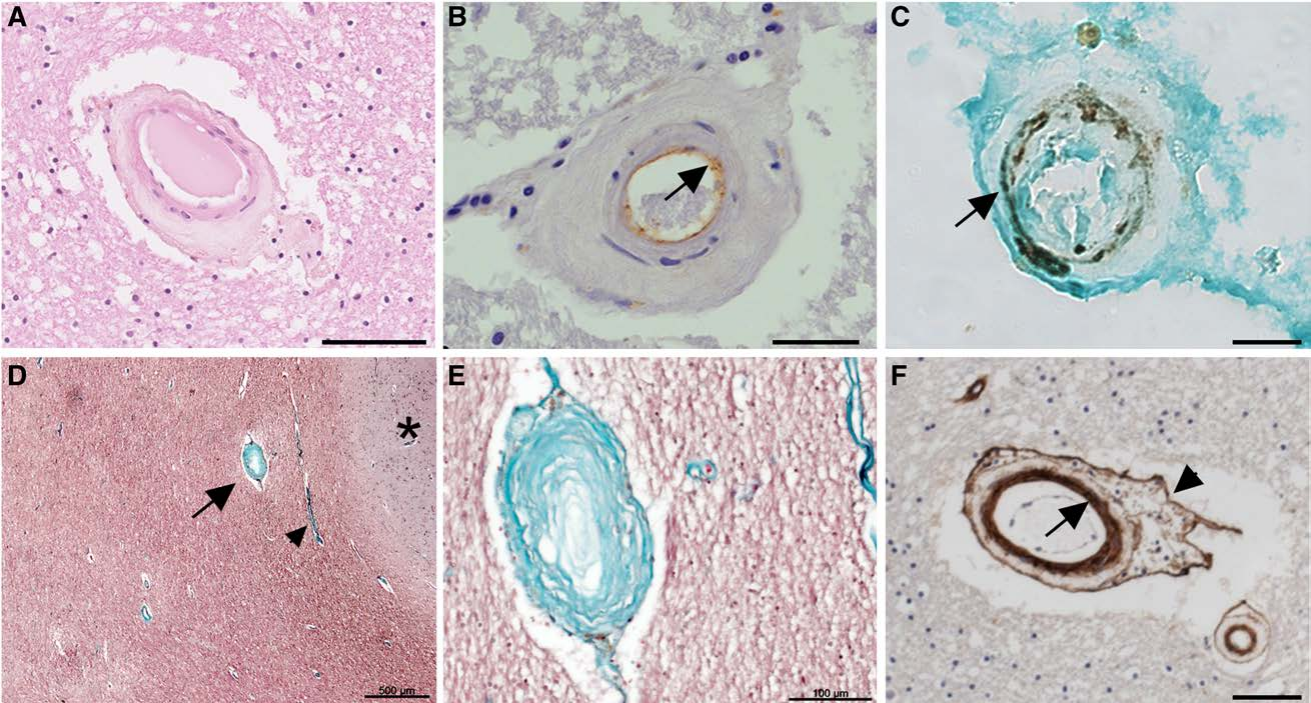
**Histopathology of arteriolosclerosis.** Small penetrating arteries in subcortical white matter. **A**, Arteriolosclerosis, with hyaline, fibrotic wall thickening, and depleted, swollen myocytes. HE stain. **B**, The endothelial cell layer is preserved, immunolabeled with thrombomodulin (brown, arrow). **C**, Depletion of myocytes, immunolabeled with smooth muscle myosin (black, arrow). Light green counterstain. **D** and **E**, Collagenous fibrosis (green) in the vessel wall, Masson trichrome stain. **D** shows a severely fibrotic vessel (arrow), close to another that exhibits no fibrosis (arrowhead), adjacent to the cortical gray matter (marked with *). In **E**, the fibrotic vessel is enlarged. **F**, Collagen type IV α1/α2 immunolabeling shows duplication of the intimal basal lamina (brown, arrow), maintained adventitial layer (arrowhead), and absence of type IV collagen in the hyaline fibrotic media. Scale bars: 50 (**A**), 20 (**B**), 50 (**C**), 500 (**D**), 100 (**E**), and 50 μm (**F**).

In arteriolosclerosis, there is a lack of lipid deposition in the walls of SVD-positive vessels (in contrast to lipohyalinosis and microatheroma^6,7^). Afflicted vessels are up to 300 microns outer diameter, characterized by concentric acellular layers of fibrous material^6,7^ (Figure 2B; Figure 3). The endothelial layer remains intact and appears paradoxically healthy^55^ alongside depletion of myocytes in the vessel wall^6,7,56^ (Figure 3B and 3C). The degree of wall thickening manifests to varying degrees along the length of a vessel.^17,57^ Luminal narrowing is not a universal feature,^58^ though striking examples are sometimes seen (Figure 3D and 3E).^57^

No serial sectioning study has been performed to test directly whether arteriolosclerosis is linked to lacune formation (or other manifestations of SVD, such as diffuse white matter damage). Based on vessel wall thickening and fibrosis, some dysfunction in CBF autoregulation is likely, making downstream hypoperfusion damage biologically plausible.^6,59^ The severity of arteriolosclerosis is increased within WMH^60^ and correlates with number of lacunes.^43^ In 1997, Lammie et al^36^ demonstrated that arteriolosclerosis was common in 70 consecutive SVD cases (whereas lipohyalinosis and microatheroma were not). Overall, we consider it likely that arteriolosclerosis is a cause of symptomatic SVD, indeed a common cause.

Immunohistochemical studies indicate that the mural fibrosis in arteriolosclerosis is due to deposition of the fibrillar collagen types I and III, not the nonfibrillar basement membrane type IV.^44^ The basement membrane components collagen-IVα1/IVα2 are genetically associated with sporadic SVD, their expression is restricted to the subendothelial region, often in multilaminar layers, and also to an adventitial layer encasing the vessel^44^ (Figure 3F). The endothelia of afflicted vessels are strongly positive for thrombomodulin (Figure 3B) indicating a highly anticoagulant lumen and ICAM1 (Intercellular Adhesion Molecule-1) negative.^61^ This is the antithesis of an activated endothelium and speaks against local vascular inflammation as a feature of arteriolosclerosis.^61^ Most neuropathology studies^62–64^ have found no association of SVD with local blood-brain barrier (BBB) dysfunction. This conflicts with some imaging findings^65–68^ and does not exclude a role for BBB changes in early phases of the disease process.

In comparison, cerebral amyloid angiopathy (CAA) is not related to hypertension or other traditional vascular risk factors (Table 2). CAA is associated with age, AD pathology, and the *APOE* e4 allele, a well-known risk factor for AD. CAA, which frequently accompanies AD-related amyloid plaques, is restricted primarily to cortical gray matter, sparing subcortical white matter^69^ (whereas lipohyalinosis and arteriolosclerosis are subcortical). Similarly, CAA is related to microinfarcts and microbleeds in the neocortex (rather than in white matter). CAA is reviewed elsewhere.^8,17^

### Parenchymal Pathology

**Figure 4.**
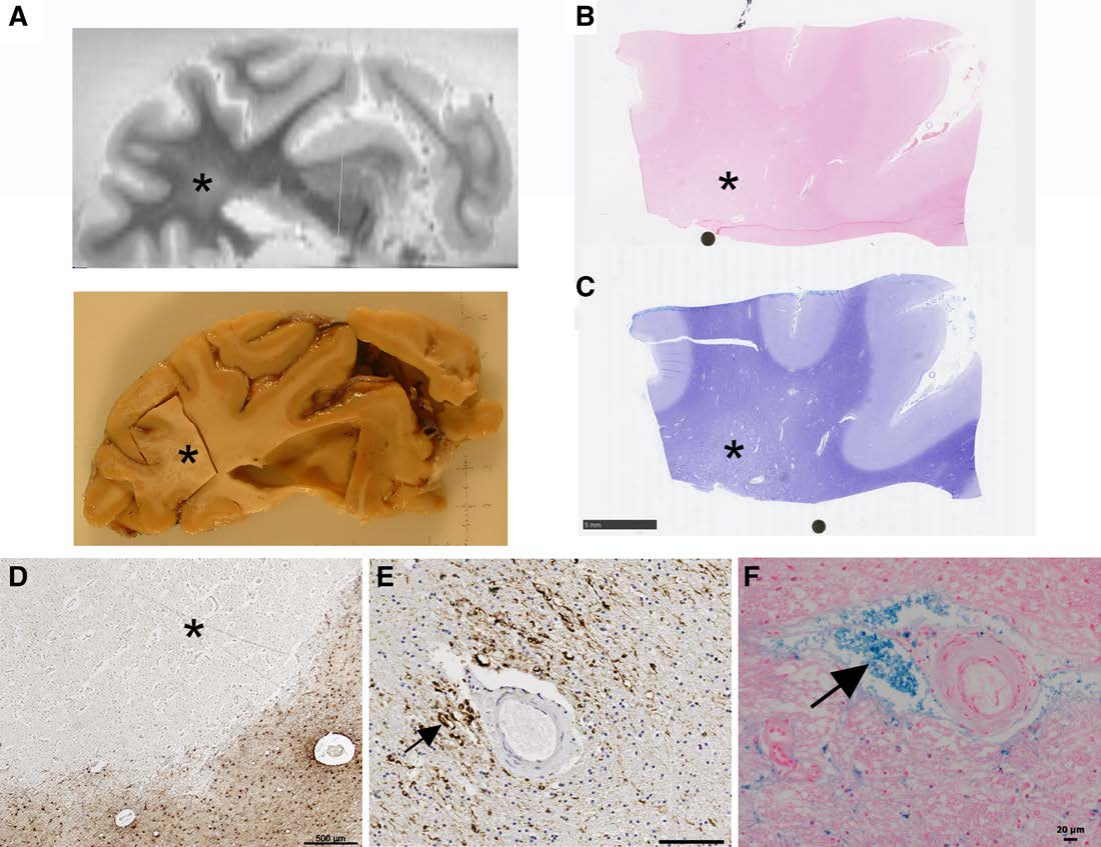
**Parenchymal lesions associated with small vessel disease. A**, Ex vivo magnetic resonance imaging-directed tissue sampling of a frontal cortical block containing white matter hyperintensity (marked with *) from the Rush archive. **B** and **C**, The block from **A**, stained with hematoxylin-eosin (**B**) and Luxol fast blue (**C**), showing white matter pallor (*). **D**, In another case, astrocytes immunolabeled with GFAP (glial fibrillary acidic protein) (brown) around the border of a microinfarct (*). **E**, Axonal bulbs (arrow), immunopositive for hyperphosphorylated neurofilament-H (brown), centered around a small penetrating artery. **F**, Microhemorrhage (marked with arrow) labeled with Perl’s stain, around a small artery. Scale bars: **B** and **C**, 5 mm. **D**, 500 microns. E, 100 microns. F, 20 microns.

While the pathological features that result from SVD are still debated, the following are generally agreed. First, small (<15 mm greatest diameter) ischemic foci or lacunes (from the Latin lacuna, meaning a hole or pond). Old lacunes can be seen as a cavity, slit, or scar, fluid-filled in vivo (Figure 1B) encased by a layer of fibrous reactive astrocytes with scattered remaining macrophages, variable microgliosis, and sparse if any lymphocytes (Figure 4D). Second, focal or diffuse white matter changes, with pallor and reduced tissue density, suggestive of edema (during acute stages) and some degree of demyelination in chronic, severe lesions (Figure 4A through 4C).^18,70,71^ These are presumed to correspond to WMH seen on MRI scans^50^ (Figure 1B). A parenchymal feature that is topologically linked with small penetrating arteries is hyperphosphorylated neurofilament-H within axonal bulbs (Figure 4E).^72^ Other features include subcortical iron deposits, some of which represent microhemorrhages (Figure 1C), but also possibly perivascular extravasation, detected in MRI scans of some older people with sporadic SVD^17^ (Figure 4F). In hypertension, microhemorrhages are usually seen in subcortical gray or white matter. Recent reviews have discussed relationships between pathological aspects of different forms of SVD and the characteristic features seen on MRI.^17,18,66^

## CLINICAL MANAGEMENT OF SVD

The most important clinical features of SVD are lacunar stroke and vascular cognitive impairment, encompassing dementia.^8,12^ We now realize that SVD is also a major cause of intracerebral hemorrhage.^8^ SVD is associated with subcortical microbleeds seen on T2* or susceptibility-weighted MRI scans^73^ and is a risk factor for subcortical intracerebral hemorrhage.^74^ Other clinical presentations include gait disturbance, apathy and other neurobehavioral symptoms, and a non-Levodopa responsive Parkinsonian syndrome. Particularly prominent features of the cognitive impairment include executive dysfunction and impaired processing speed.^75^

### BP Control, SVD, and VCID

Intensive BP lowering as a strategy to reduce risk of cognitive impairment is supported by several clinical studies.^76,77^ Data from over 9000 older Americans indicated that this effect was most evident in the oldest-old and was not driven by any specific drug class.^77^ In other words, reduced systemic pressure was sufficient to produce cognitive benefit. The small but significant reduction in WMH progression suggests that this effect is accompanied by amelioration of SVD.^78^

Despite its public health importance, there are few specific treatments for SVD.^79,80^ Most of the clinical trials looking at secondary prevention of stroke have not adequately subtyped SVD to allow us to know how useful they are in SVD. The one exception is blood pressure, for which clinical trial data show that intensive treatment reduces the risk of further stroke and probably also the risk of vascular cognitive impairment^81^ and that this strategy appears to be safe in patients with severe SVD in whom autoregulation may be affected.^82^ Epidemiological evidence suggests that there is a greater benefit from treating hypertension in midlife than in later life, particularly for the prevention of dementia.^83^ Antiplatelet agents are widely used in SVD although there are limited data on which to base this decision. A multi-center phase-3 trial demonstrated that aspirin alone was preferable to dual antiplatelet therapy with aspirin and clopidogrel in long-term secondary prevention.^84^

Two major factors underlie our lack of knowledge about treatments in SVD and VCID.^79,80^ The first has been inadequate knowledge about the underlying pathophysiology and potential treatment targets. This situation is improving with genetic studies and other advances.^14,79^ The second is a lack of adequate subtyping in clinical trials, to determine whether treatments are efficacious in definite SVD. Recent phase II clinical trials have tested existing medications for possible repurposing in people with symptomatic SVD. These include the PDE5 (phosphodiesterase-5) inhibitor tadalafil^85^ and the combination of cilostazol with a NO donor.^86^ Sets of criteria for the definition of SVD for clinical trials, and for finding targets for intervention, have been published.^87,88^ Recent guidelines have been developed to provide a framework for optimal trial design in SVD, including choice of outcome measures.^89^

### Genetics of SVD

In discussing genetic factors in sporadic SVD, it is instructive to consider monogenic, familial forms of SVD. A number of different underlying genes have been described but by far the most common is CADASIL, caused by *NOTCH3* mutations. The second most common is CADASIL2 caused by autosomal dominant *HTRA1* mutations.^90^ *HTRA1* is also the gene underlying CARASIL (with autosomal recessive inheritance). Major clinical features of both CADASIL and CADASIL2 include migraine with aura, early-onset lacunar stroke, encephalopathy, depression, and early-onset dementia. MRI features are similar to those of sporadic SVD but WMH are often severe at a younger age and frequently seen in the anterior temporal poles and the corpus callosum, which are rarely affected in sporadic SVD. Microbleeds occur in both CADASIL and CADASIL2 but are particularly frequent in the *COL4A1/2* monogenic forms of SVD, which also frequently present with intracerebral hemorrhage.^91^

Genetic risk is also important for sporadic SVD, but here common variants or polymorphisms in multiple genes are thought to each confer a small increase in risk. To date, over 50 independent genetic loci have been associated with SVD at the genome-wide significance level, including loci associated with lacunar stroke^13^ and with covert, MRI-defined SVD.^14,15^ The genes identified point to a major role of blood pressure–related pathways, and also mechanisms that seem independent of vascular risk factors, particularly ECM (extracellular matrix) structure and function. Future use of high-throughput molecular approaches (epigenomics, transcriptomics, proteomics, and metabolomics) will enable integration of genetic associations with functional data to decipher the biological roles of genetic risk loci in SVD.^14^

Genetic studies in sporadic SVD are highlighting novel disease processes and potential therapeutic pathways. A key target appears to be the ECM and proteins of the matrisome,^92^ defined as the ensemble of 1000+ genes encoding ECM-associated proteins. *NOTCH3* and *HTRA1* mutations seem to cause ECM disruption by converging pathways, while *COL4A1/2* mutations result in disruption of the collagen-IV matrix essential for ECM integrity.^92^ Increasing evidence suggests monogenic and sporadic SVD share disease mechanisms; common variants in both *HTRA1* and *COL4A1/2* have been associated with both sporadic lacunar stroke and WMH.^13,15^

The boundaries between sporadic and monogenic SVD have been blurred by the finding that typical mutations causing monogenic SVD seem to be much more common in the general population than expected from the prevalence of clinical monogenic SVD.^14^ For example, while the reported prevalence of CADASIL is about 4 in 10 000 in the United Kingdom, typical cysteine-changing *NOTCH3* mutations are seen in 1 in 800 individuals in a large population cohort (UK Biobank).^93^ A similar situation was found for *HTRA1* and *COL4A1/2* mutations.^93^ The clinical significance of these mutations in the general population remains to be determined but their presence is associated with an increased risk of stroke and VCID.^93^ Why some mutations result in typical monogenic disease and others are much less penetrant is uncertain, although cardiovascular risk factors and mutation position influence their phenotype,^93^ and modifier genes have been hypothesized.

## PRECLINICAL MODELS IN SVD AND VCID

Experimental animal models relevant to SVD and VCID have been reviewed by ourselves and others.^94–96^ Spontaneous lesions occur in some rodent models, for example, spontaneously hypertensive stroke-prone rats develop focal infarcts and hemorrhages.^97^ Spontaneously hypertensive stroke-prone rats do not display the vessel pathology of SVD^44,98^ and chronic hypertension alone appears insufficient to generate SVD-like arterial changes in rats or monkeys.^44,98^ Molecular pathways link hypertension, endothelial dysfunction, and oligodendrocyte damage in spontaneously hypertensive stroke-prone rats.^99^

In our view, rodent models offer a platform to assess some specific aspects of SVD pathophysiology (such as blood vessel fibrosis, perivascular trafficking, white matter degeneration) but do not provide a unified model of the whole disease process occurring in humans.^94,95^ Larger species (primates, canines, ovines, swine) have the advantages of a large gyrencephalic brain and cerebral blood vessels that more resemble their human counterparts.^44,94^

## HYPOTHESES OF SVD PATHOGENESIS

The mechanism by which SVD vascular pathology leads to parenchymal changes remains incompletely defined. Mechanisms may be heterogeneous and dependent on the risk factor profile (severity of hypertension, diabetes), the type of SVD, and its anatomic location.^3^ Various pathogenic pathways have been proposed.

First, an early, simple hypothesis was that, by analogy with large vessel ischemic stroke, small artery embolic occlusion produced a correspondingly smaller, lacunar lesion.^100^ This was not supported by the serial sectioning of brain tissue performed by Fisher^5^ who found few emboli, or by transcranial Doppler imaging in lacunar patients with stroke where embolic events were rare.^101^ As small arteries exhibiting SVD have abundant endothelial thrombomodulin, the potent physiological antithrombotic protein, local thrombosis leading to occlusion appears unlikely.^61^

In a second hypothesis, local BBB dysfunction causes SVD vessel pathology, as well as parenchymal changes.^36,102^ The rationale is that multiple reactive, harmful plasma components are allowed access to the CNS, triggering cell damage in the artery wall and diffuse myelin and axonal damage in the white matter. Plasma extravasation also represents an excess water load, with risk of vasogenic edema. In support of this view, the WMH attributed to SVD resembles fluid diffusion patterns. MRI data show enhanced brain accumulation of intravascular contrast agents in people with SVD, relative to controls,^65^ with accumulation especially evident within WMH.^65,67,68^ By contrast, neuropathology studies have not supported this hypothesis, with extravascular plasma markers showing little relation to SVD.^62–64^ The relation of histological markers of BBB dysfunction to AD pathology is more robust.^103^

In a third hypothesis, chronic underperfusion (oligemia) links small artery fibrosis to diffuse white matter damage and ultimately a focal lacunar lesion. In support of this view, regulation of cerebral blood flow—rapidly and precisely autoregulated in healthy brain tissue—is disrupted in lacunar stroke.^104,105^ This accords with neuropathological findings that histological markers of cell ischemia are expressed within WMH.^60^ Insufficient arteriolar dilatation, leading to repeated episodes of underperfusion, might be expected to cause cumulative damage in brain tissue, with lesional features including disruption of myelin structure, some axonal loss, patchy BBB dysfunction, congregation of microglia and macrophages (to remove debris), and heightened astrocyte activity (to clear edema fluid). These features resemble those of an incomplete lacune.^106^ As oligemia, edema, and BBB dysfunction are all interdependent, separating this from the previous hypothesis is challenging, especially in a chronic, nonfatal disease.^59^

Fourth, a further suggested pathogenic process is inflammation, both systemic and within the CNS.^4^ Inflammatory cells are found in the white matter of some postmortem brains from patients with SVD.^60,64,107^ Evidence for CNS inflammation is also supported by positron emission tomography imaging of patients with SVD, using radioligands such as 11C-PK11195 targeted against the translocator protein, a mitochondrial surface protein upregulated in microglial activation. Both increased global 11C-PK11195 binding^108^ and focal hot spots of increased binding, have been reported in SVD.^109^ Systemic blood markers of inflammation have also been reported to be elevated in SVD and to correlate with radiological disease severity, although whether such changes are casual or secondary to tissue damage is uncertain.^110,111^

### Possible Molecular Mechanisms

In terms of molecular mechanisms, preclinical data from various laboratories have suggested possible hypotheses as to how hypertension influences neurovascular function and VCID.^112^ Invoking neuroinflammation, endothelial dysfunction, and fibrosis, these mechanisms include aberrant activity of the renin-angiotensin system, loss of BBB components, or defective NO, cytokine, or endothelin signaling. Examples of particular interest include IL-6 (interleukin-6),^113^ ET-1 (endothelin-1) (via ET_A_ receptors),^114^ and perivascular collagen production.^44,115^ The dramatis personae of the neurovascular unit, including pericytes, myofibroblasts, and perivascular macrophages^116^ are all likely participants.^117^

## SUMMARY AND FUTURE DIRECTIONS IN SVD AND VCID

This is an exciting time in SVD research. The dementia field in general has been energized by recent progress in antibody therapy for AD, which will have spin-off benefits for VCID research.^118^ Early-phase clinical trials in SVD are also emerging.^79,85,86^ Though neutral, these offer possible directions for further trials to deliver therapeutic interventions in SVD and hence in VCID.^80^ We anticipate more, carefully designed clinical trials in SVD, utilizing recently developed guidelines.^87–89^

It is a truism that since the era of C. Miller Fisher this field has lacked molecular targets. Modern biology and big data streams are changing that. Genome-wide association study data have provided an impressive catalog of candidate genes linked to sporadic SVD.^14^ All will require conscientious laboratory studies to evaluate their significance in disease. At the level of gene expression, there is a burgeoning collection of cell atlases derived from brain tissue samples.^117^ These reveal disease-related changes in the RNA profile of each cell type, including vascular cells, within a brain biospecimen.^117,119,120^ Coupled with advances in single-cell proteomics, these atlases are informing on cell subtypes (eg, subfamilies of oligodendroglia, microglia, and endothelial cells) and cell-cell interactions relevant to brain disease. For SVD, they will reveal novel molecular agents as possible biomarkers and drug targets.^79,80^ Aided by novel chemistry and better translational platforms, all accelerated by the power of artificial intelligence, these are likely to provide real progress in how we understand and treat SVD and VCID.

## ARTICLE INFORMATION

### Acknowledgments

The authors thank Fatemeh Geranmayeh, Gustavo Roman, and Colin Smith for comments on the article. A.H. Hainsworth thanks Leslie Bridges, Margaret Esiri, and Alistair Lammie for many helpful discussions. The views expressed in this article are those of the individual authors.

### Sources of Funding

Research in A.H. Hainsworth’s group is funded by the UK Medical Research Council (MR/R005567/1 and MR/T033371/1), British Heart Foundation (PG/20/10397 and SP/F/22/150042), and UK Alzheimer’s Society and Alzheimer’s Drug Discovery Foundation (20140901). H.S. Markus is supported by a British Heart Foundation program grant (RG/F/22/110052). Infrastructural support was provided by the Cambridge British Heart Foundation Centre of Research Excellence (RE/18/1/34212) and Cambridge University Hospitals National Institute for Health and Care Research (NIHR) Biomedical Research center (BRC-1215-20014). J.A. Schneider has received funding from US National Institutes of Health (R01AG017917, R01AG064233, R01AG061028, P30AG072975, and P30AG010161).

### Disclosures

A.H. Hainsworth has received honoraria from Eli-Lilly and from National Institute on Aging (NIA). He serves as a consultant for AriBio Co, Ltd. and chairs the DementiasPlatform UK Vascular Experimental Medicine group. J.A. Schneider has received honoraria from Eli Lilly, Alnylam Pharmaceuticals, Inc, Apellis Pharmaceuticals, Inc, and Fondation Alzheimer. She has served as an expert witness for the US National Hockey League. The other author reports no conflicts.
